# An Untargeted Metabolomics Approach to Investigate the Metabolic Effect of Beetroot Juice Supplementation in Fencers—A Preliminary Study

**DOI:** 10.3390/metabo10030100

**Published:** 2020-03-11

**Authors:** Lucyna Kozlowska, Olga Mizera, Anna Mroz

**Affiliations:** 1 Department of Dietetics, Faculty of Human Nutrition, Warsaw University of Life Sciences—WULS, 02-776 Warsaw, Poland; 2 Department of Physiology and Sport Medicine, Jozef Pilsudski University of Physical Education in Warsaw, 00-968 Warsaw, Poland; anna.mroz@awf.edu.pl

**Keywords:** beetroot juice, VO_2max_, urine metabolites, neurotransmitters, 4-Hydroxynonenal

## Abstract

This study aimed at assessment of the long-term (4 weeks) metabolic effect of a diet with and without beetroot juice supplementation in fencers using the untargeted metabolomics method with the UPLC Q-TOF/MS system to carry out an analysis of urine samples. Ten women and 10 men underwent the cardiovascular fitness VO_2max_ test at baseline—(B) and after two stages of implementation of the dietary recommendations—the first 4 weeks without beetroot juice (D) and the second with 26 g/d of freeze-dried beetroot juice supplementation (D&J). The urine samples were collected one hour after the VO_2max_ test at B and after D and D&J. The meal before the VO_2max_ test after D&J contained beetroot juice, whereas to the meal at B and after D maltodextrin was added. Changes in metabolites and VO_2max_ were significant only for comparison of D versus D&J. During D and D&J, there were no significant changes in the physical activity level, body mass, and body composition. We observed significant changes in tyrosine and tryptophan metabolism, mainly associated with such neurotransmitter’s metabolism as: Serotonin, noradrenaline, and adrenaline. Changes in signal intensity of bile acid, AICAR, and 4-Hydroxynonenal (peroxidation of polyunsaturated fatty acids product) were also observed. The obtained results indicate that long-term beetroot juice supplementation induces considerable changes in metabolism.

## 1. Introduction

Beetroot (*Beta vulgaris var. rubra*) juice is a source of carbohydrates, fiber, proteins, and minerals (sodium, potassium, calcium, and iron) as well as other bioactive compounds, including: Phenolic acids, flavonoids, and phenolic amides with betalains [1]. Additionally, the juice has high inorganic nitrate contents, and due to the physiological effect of this ingredient, it is used as a supplement [2]. Benjamin et al. [3] and Lundberg et al. [4] have noted that dietary nitrate can be a source of gastric nitric oxide. These authors investigated only the local biological processes without the analysis of a systemic source of nitric oxide. The formation of nitric oxide from dietary nitrite and nitrate is a matter of debate for about 20 years. The latest deep review on the nitrate-nitrite-nitric oxide pathway has described key steps in the elucidation of this pathway. In brief, reduction of nitrate to nitrite takes place on the posterior surface of the tongue, after which nitrite is absorbed from the upper gastrointestinal tract into the circulation. Subsequently, nitrite undergoes a further reduction to produce biologically active nitric oxide [5]. This important compound can determine many physiological functions such as: Vasodilatation, blood flow and oxygen volume regulation, mitochondrial biogenesis, mitochondrial respiration, glucose uptake, and muscle contraction or relaxation [6,7]. Intensification of nitrite anion reduction during hypoxia is a very important process that leads to the increased generation of nitric oxide in those parts of the muscle that need more oxygen. This mechanism would also improve local blood flow and delivery of oxygen within working skeletal muscles. Additionally, nitric oxide is known to be an important inhibitor of cytochrome oxidase activity [8] and might enhance the efficiency of oxidative phosphorylation [9]. In the presence of nitrite, pentacoordinate cytochrome *c* generates bioavailable nitric oxide that is able to inhibit mitochondrial respiration [10]. Due to the above-mentioned metabolic effects, dietary nitrate has been commonly investigated with respect to the improvement of physical performance. In many studies, it has been shown that beetroot juice supplementation can improve performance at various distances, increase time to exhaustion at submaximal intensities and that it may improve cardiorespiratory performance at anaerobic threshold intensities and maximum oxygen uptake [7]. However, in highly-trained athletes, performance gains seem harder to obtain [2]. Bearing in mind the quite well-known physiological acute and chronic (up to 15 days) effects of dietary nitrate, the International Olympic Committee classified it as a supplement with good to strong evidence of bringing benefits to performance when used in specific scenarios [11]. 

However, there are no studies on which nitrates would be administered to highly trained athletes on a long-term basis, and there are no studies that would analyze the wide metabolic effect of such an intervention. Therefore, the aim of our study was to analyze the long-term (4 weeks) metabolic effect of a diet with and without beetroot juice supplementation in fencers (women and men competing at the international level) using the untargeted metabolomics method to carry out an analysis of urine samples taken after the cardiovascular fitness test. 

## 2. Results

### 2.1. Anthropometric Parameters, Physical Activity Level and Cardiovascular Fitness 

Our study cohort consisted of 10 females and 10 males. Body weight, body mass, and body fat remained stable during the intervention (Table 1). In addition, the mean physical activity level in both groups of fencers was at the same level, with the exception of fencing training days in the men, when it was slightly higher at baseline (B) versus the stage with the implementation of the dietary recommendations (D). Nevertheless, during the whole period of the nutritional intervention, there were no significant differences between an activity level during D and during the stage with the implementation of the dietary recommendations and beetroot juice supplementation (D&J) on this kind of training days. In the case of cardiovascular fitness measured using VO_2max_ in both groups of fencers, no significant differences were observed between the mean values at B and after D. However, after 4 weeks of D&J versus D in both groups of fencers, a significant increase in the mean value of VO_2max_ was observed (Table 1).

During the first and second stages of the study, the dietary intake of the selected nutrients was monitored. Overall, no significant changes were observed in the group of women and men in the dietary intake of the selected nutrients with three exceptions—in the men during D&J in comparison with D, energy and fat intake was significantly higher. Additionally, vitamin B_12_ was lower by an average of 93 kcal/day, 7.3 g/day, and 1.19 µg/day, respectively. Despite the decrease in vitamin B_12_ intake, its mean consumption during D&J in the men was still about three times higher than the recommended dietary allowance for this group of people. In the group of women, there were no significant changes in energy and the selected nutrients intake between D and D&J (Table 2). 

### 2.2. Untargeted Metabolomics Studies

**Figure 1:**
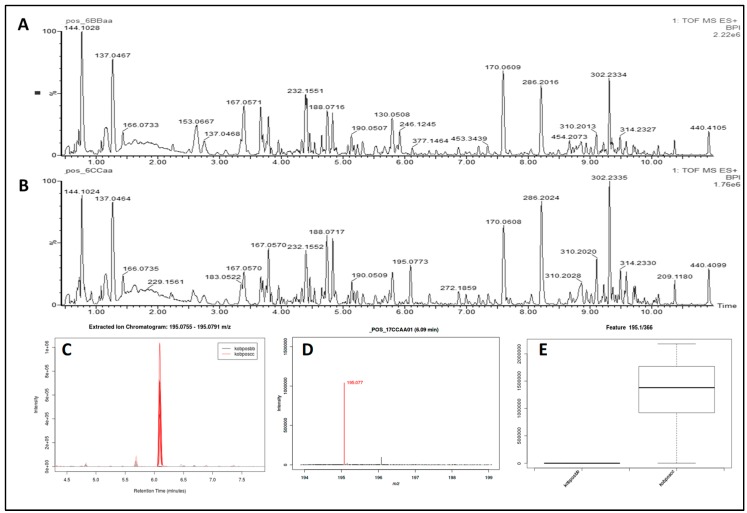
**A**–**E.** Exemplary UPLC-QTOF MS profiling in ESI+ mode and results obtained by means of the XCMS Online. (**A**) The base peak intensity chromatogram (BPI) of a urine sample of a woman after implementation of the dietary recommendations; (**B**) BPI chromatogram of a urine sample of a woman after implementation of the dietary recommendations and beetroot juice supplementation; (**C**) the extracted ion chromatogram: 195.0755–195.0791 *m/z*; (**D**) the mass spectrum at 6.09 min selected to highlight the peak for 195.0772 *m/z*, which represent the M+K[1+] species; (**E**) the box-and-whisker plot for that metabolic feature.

The representative based peak intensity (BPI) chromatograms of the urine samples from one woman after stages D and D&J in ESI+ mode obtained using the optimal UPLC Q-TOF/MS conditions, the extracted ion chromatogram, the mass spectrum, and the box-and-whisker plot are presented in Figure 1A–E.

**Figure 2:**
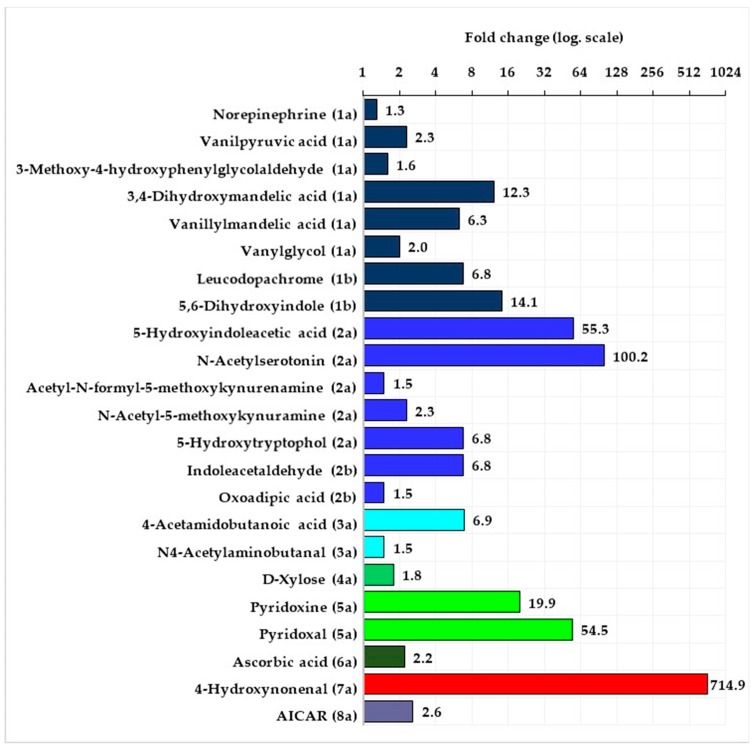
Mean fold change of intensity signal from the tentatively identified compounds belonging to the specific pathways (the numbers in brackets specify the subpathways name) in the group of women after the stage with the implementation of the dietary recommendations and beetroot juice supplementation versus after the stage with the implementation of the dietary recommendations.

**Figure 3:**
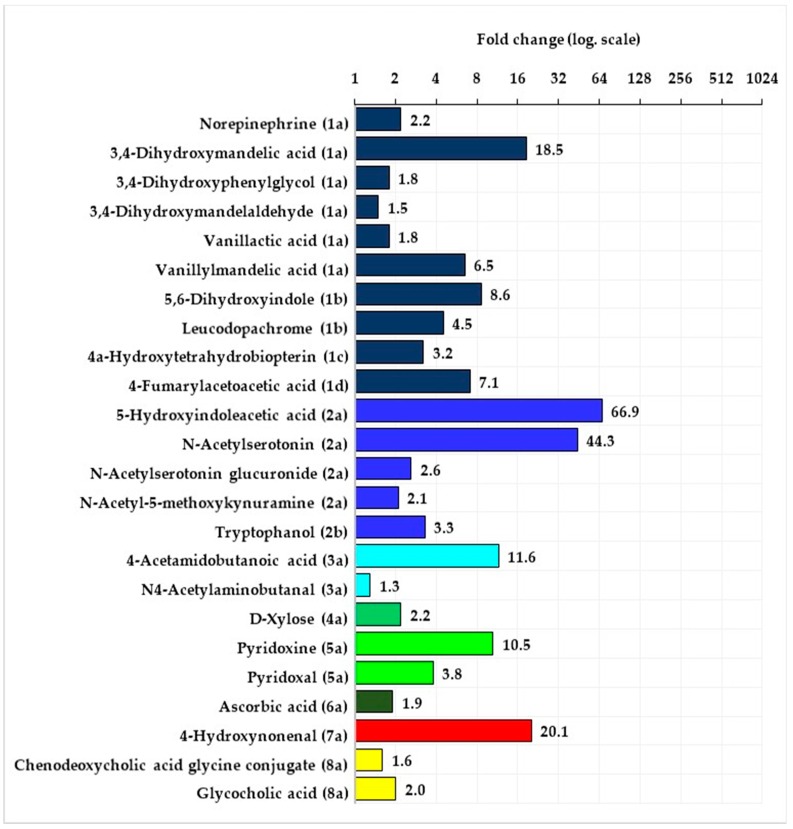
Mean fold change of intensity signal from the tentatively identified compounds belonging to the specific pathways (the numbers in brackets specify the pathways name) in the group of men after the stage of implementation of the dietary recommendations and beetroot juice supplementation versus after the stage with the implementation of the dietary recommendations.

The statistical analysis of the data obtained by means of the XCMS-online program showed that in the women as well as in the men, there were no significant differences between signal intensity of features in the urine samples taken at B versus those taken after D. Significant differences in signal intensity of tentatively identified metabolites were observed in both groups of fencers in the samples taken after D&J versus those taken after D. In both groups—of the women and men signal intensity from all significantly changed compounds was higher after D&J in comparison with after D. These compounds in the women and men belonged to the following pathways: metabolism of tyrosine, tryptophan, arginine, and proline, vitamin B6, ascorbate and alderate, peroxidation of lipids as well as interconversions of pentose and glucoronate. Additionally, in the women, a compound from the purine metabolism pathway and in the men—compounds from bile acid biosynthesis was significantly changed (Figure 2 and Figure 3, Supplementary materials Tables S1 and S2). 

The majority of the tentatively identified compounds came from tyrosine and tryptophan metabolism pathways. The compounds from tyrosine metabolism belonged to the subpathways of noradrenaline and adrenaline degradation (6 metabolites in both men and women) as well as L-dopa and L-dopachrome biosynthesis (2 metabolites in both men and women). Additionally, in the group of men compounds belonging to subpathway conversion of phenylalanine to tyrosine and L-tyrosine degradation was found. Fold change compounds from tyrosine metabolism pathway in the groups of women and men were at a similar level and ranged from 1.3 to 14.1 in the women and from 1.5 to 18.5 in the men. With regard to the tryptophan metabolism pathway, significant changes in signal intensity from metabolites belonging to two subpathways (serotonin and melatonin degradation, L-tryptophan degradation) were observed. There were 5 metabolites in the women and 3 in the men from serotonin and melatonin degradation and 2 metabolites in the men as well as in the women from L-tryptophan degradation. In the case of this pathway, the biggest changes were observed in signal intensity from 5-Hydroxyindoleacetic acid and from N-Acetylserotonin—fold change in the women of above 50 and 100, in the men of more than 60 and 40, respectively. Fold change of other metabolites belonging to the tryptophan pathway was in the range of 1.5 to 6.8 in the women and 2.1 to 3.3 in the men.

Tyrosine metabolism: (1a)—subpathway noradrenaline and adrenaline degradation, (1b)—subpathway L-dopa and L-dopachrome biosynthesis. Tryptophan metabolism: (2a)—subpathway serotonin and melatonin degradation, (2b)—subpathway L-tryptophan degradation. Arginine and proline metabolism: (3a)—subpathway putrescine degradation III. Pentose and glucuronate interconversions: (4a)—subpathway D-xylose degradation I. Vitamin B6 metabolism: (5a)—subpathway pyridoxal 5’-phosphate salvage. Ascorbate and alderate metabolism: (6a)—subpathway ascorbate recycling. Lipid peroxidation: (7a)—subpathway 4-hydroxy-2-nonenal detoxification. Purine metabolism: (8a)s—subpathway purine nucleotide biosynthesis. 

Tyrosine metabolism: (1a)—subpathway noradrenaline and adrenaline degradation, (1b)—subpathway L-dopa and L-dopachrome biosynthesis, (1c)—subpathway conversion of phenylalanine to tyrosine, (1d)—subpathway L-tyrosine degradation I. Tryptophan metabolism: (2a)—subpathway serotonin and melatonin degradation, (2b)—subpathway L-tryptophan degradation. Arginine and proline metabolism: (3a)—subpathway putrescine degradation III. Pentose and glucuronate interconversions: (4a)—subpathway D-xylose degradation I. Vitamin B6 metabolism: (5a)—subpathway pyridoxal 5’-phosphate salvage. Ascorbate and alderate metabolism: (6a)—subpathway ascorbate recycling. Lipid peroxidation: (7a)—subpathway 4-hydroxy-2-nonenal detoxification. Primary bile acid biosynthesis: (8a)—subpathway bile acid degradation.

In both groups of the women and men, the same metabolite belonging to the subpathways of putrescine degradation III, D-xylose degradation I, pyridoxal 5’-phosphate salvage, ascorbate recycling and 4-hydroxy-2-nonenal detoxification were significantly changed. However, in the case of vitamin B_6_ metabolites and metabolites of lipid peroxidation product (4-Hydroxynonenal) fold changes in signal intensity after D&J versus D were many times higher in the women when compared to the men. Additionally, after D&J vs. D in the women there was a more than two times higher fold change from metabolite of subpathway purine degradation was observed, and in the men, there was about twice higher fold change from two metabolites of the subpathway bile acid degradation.

## 3. Discussion

To our knowledge, this is the first untargeted metabolomics study created for the purpose of an analysis of metabolism changes after using a long-term diet with and without beetroot juice supplementation in high-trained athletes. The study included highly trained female and male fencers. It was planned in such a way that during the whole 8 weeks of observation, the fencers would not change their dietary intake and physical activity. During the whole study period, no significant changes were observed with respect to body mass and body composition. It was also taken care thus that the meals given to the athletes 2 h before the VO_2max_ test consisted of the same products and had the same weight and energy value (in order to balance the energy value, at B and after D maltodextrin was added to the meal—26 g and after D&J freeze-dried beetroot juice was added to it—26 g). After 4 weeks of D in comparison to B, no significant changes were observed with regard to the mean values of the maximal oxygen consumption VO_2max_ test. In addition, there were no significant changes between signal intensity of metabolites coming from the urinary samples taken 1 h after VO_2max_ test at B and after D. Whereas after the next 4 weeks, during which the fencers still did not change their dietary intake and physical activity but had the freeze-dried beetroot juice (26 g/day) supplemented a significant increase in the VO_2max_, as well as changes in signal intensity coming from urinary metabolites, were observed. Most changes were observed in amino acid metabolism, such as: Tyrosine, tryptophan, arginine, and lipid peroxidation. Additionally, compounds coming from xylose, purine, bile acids, vitamins B_6,_ and C metabolism were also significantly changed. Thus far, studies using dietary nitrate have focused mostly on such physiological functions as: Vasodilatation, blood flow and volume of oxygen regulation, mitochondrial biogenesis, mitochondrial respiration, glucose uptake, and muscle contraction or relaxation [7]. Our studies indicate that the metabolic effect of the administered to the fencer’s beetroot juice seems to be more complex. 

In a mammalian organism, nitric oxide is synthesized endogenously in several types of cells, such as neurons, endothelial cells, and macrophages by a family of three isoenzymes termed nitric oxide synthases, which utilize L-arginine as a substrate [12]. Functions and metabolic effects of endogenously synthesized nitric oxide have been fairly well studied. It has been shown that endogenously synthesized nitric oxide not only operates as an intercellular messenger but additionally diffuses rapidly and influences nitric oxide responsive target cells. Therefore, a released nitric oxide may influence neurons in an extended area. [13]. Nitric oxide is a messenger molecule, which has numerous molecular targets, among others control of servo-regulatory functions such as neurotransmission [14]. With respect to the modulation of neuronal function by nitric oxide studies in vivo and in vitro have shown that, in all brain structures, nitric oxide modulates the release of several neurotransmitters. Under in vitro and in vivo conditions, nitric oxide donors increase the release of noradrenaline in the hippocampus [15,16]. Additionally, the in vitro release of noradrenaline, stimulated by N-methyl-D-aspartate or 3,4-diaminopyridine is enhanced by nitric oxide donors, while nitric oxide synthase inhibitors exert the opposite effect [17]. In the medial preoptic area [18] and the striatum [19], serotonin release is enhanced by L-arginine and nitric oxide donors, respectively. It has also been shown that nitric oxide donors in the hypothalamus and in the locus coeruleus enhance the release of serotonin. What is more, nitric oxide also has a modulatory role—high nitric oxide levels increase serotonin levels in the hypothalamus, while low nitric oxide concentrations seem to reduce them [20]. Bearing in mind the mentioned above metabolic effects of endogenously synthesized nitric oxide, it can be assumed that nitric oxide originating from a dietary nitrate may also have similar effects. In our studies, significant changes in signal intensity coming from metabolites of tyrosine and tryptophan, in particular from the subpathway of noradrenaline, adrenaline, and serotonin metabolism, as well as lipid peroxidation, were observed.

In the tyrosine metabolism pathway, most of the tentatively identified metabolites come from the subpathway of noradrenaline and adrenaline degradation. In brief, L-tyrozine is converted to L-dopa and subsequently to dopamine with pyridoxal phosphate as a cofactor. Dopamine acts as a precursor in noradrenaline and adrenaline synthesis [21]. In neurons that use dopamine as a transmitter, no further enzymatic modification occurs, but neurons that use noradrenaline as a transmitter contain an additional enzyme that converts dopamine to noradreanaline and oxygen, as well as ascorbic acid, are cofactors in this process. Neurons using adrenaline as a transmitter contain an additional enzyme, which is responsible for catalyzing the conversion of noradrenaline to adrenaline [22]. In the tyrosine metabolism pathway in both groups of fencers, there were also two metabolites from L-dopa and L-dopachrome biosynthesis pathways (Leucodopachrome and 5,6-Dihydroxyindole). Additionally, in the men 4a-hydroxytetrahydrobiopterin, which is essential for catalyzing the conversion of phenylalanine into tyrosine was identified [23] as well as an intermediate product 4-Fumarylacetoacetic acid, which belongs to L-tyrosine degradation to fumarate pathway and after that utilization in tricarboxylic acid cycle. 

With respect to tryptophan metabolism, most of the tentatively identified metabolites, as well as most of the fold changes, come from serotonin and melatonin degradation. Tryptophan conversion into serotonin occurs in the following steps: Conversion of tryptophan into 5-hydroxytryptophan by tryptophan hydroxylase and decarboxylation of 5-hydroxytryptophan into serotonin. The last step is vitamin B_6_ dependent [24]. Serotonin, among others, may be converted to 5-Hydroxyindoleacetic acid and N-Acetylserotonin and fold changes of both these metabolites in the women and men were the biggest among other metabolites from the tryptophan transformation pathway. In the next step, N-Acetylserotonin may be converted to melatonin and after that to acetyl-N-formyl-5-methoxykynurenamine (AFMK), which is a direct precursor of N-Acetyl-5-methoxykynuramine (AMK). AFMK is a product of melatonin metabolization in the brain [25]. Other metabolites coming from subpathways of tryptophan metabolism tentatively identified in our study include: L-tryptophan degradation to tryptophanol via indoleacetaldehyde and additionally, oxoadipic acid, which is an intermediate metabolite of the tryptophan conversion to Acetyl-CoA and utilization in tricarboxylic acid cycle [24]. In our study, we also observed high-intensity signal from vitamin C and B_6,_ which as cofactors take part in noradrenaline and serotonin synthesis, respectively. 

Significantly higher-fold change of serotonin can also be connected with a function of this compound as a gastrointestinal motility regulator. In the group of men who participated in our study, we also observed significantly increased signal intensity from bile acid metabolites—chenodeoxycholic acid glycine conjugate and glycocholic acid. It has been estimated that about 95% of serotonin is found in the gastrointestinal tract, of which about 90% is present in enterochromaffin cells and 10% in enteric neurons but the remainder of serotonin (5%) is found in the brain [26]. Serotonin can make the bowel contract or relax. It can stimulate cholinergic neurons to release acetylcholine, which results in a smooth muscle contraction or it can stimulate inhibitory nitrergic neurons to release nitric oxide, which results in smooth muscle relaxation. Additionally, enteric serotonin plays multiple roles acting as a paracrine factor, endocrine hormone, neurotransmitter and growth factor. It is also important in enteric neurogenesis, mucosal growth/maintenance, intestinal inflammation, osteogenesis and hepatic regeneration [27]. Morville et al., [28] in their study involving healthy, moderately trained males, have observed that total plasma bile acids decreased exclusively following resistance exercise, but the composition of bile acids changed in response to both types of exercise. Among others, after both types of exercise (60 min), serum concentrations of total, as well as unconjugated chenodeoxycholic acids, were significantly lower in comparison to the baseline condition. Bearing in mind these results, the higher amount of these two cholic acids in urine in our study may be a result of their higher elimination from the body. 

Bile acids (BAs) are not only essential factors in lipid metabolism, but they can also participate in the regulation of energy metabolism [29,30,31,32]. It has been shown that bile acids activate the TGR5 G protein-coupled receptor, which results in the increased concentrations of the cAMP, activation of type 2 deiodinase, which converts the inactive thyroid hormone thyroxine to active 3-5-3′-triiodothyronine and leads to an increased brown adipose tissue activity and enhanced energy expenditure in murine brown adipose tissue and human skeletal muscle. Indeed, the primary bile acid, cholic acid, increases whole-body energy expenditure in mice [32,33]. In a human study on a group of healthy volunteers and patients with liver cirrhosis, a positive correlation between circulating bile acids and energy expenditure has been observed [34]. Broeders et al. [35] have shown that administration of chenodeoxycholic acid in cultured adipocytes from the human brown adipose tissue region activated brown adipose tissue via a TGR5-dependent mechanism and the increase in bile acid was accompanied by increased energy expenditure. In addition, in our study, the significantly higher level of some bile acids could be an activating factor of brown adipose tissue and an additional source of energy substrates for working muscles. 

In addition, the same metabolites coming from arginine degradation were tentatively identified in both the women and men. Arginine can be converted by arginase to ornithine and after that by ornithine decarboxylase to putrescine. At a later stage of changes, among others, 4-Acetamidobutanoic and acid N4-Acetylaminobutanal, which were identified by us, were formed. These compounds are transformed into 4-Amino butanoate and enter the tricarboxylic acid cycle [36]. 

Only in the group of women, AICAR (5-amino-imidazole carboxamide riboside)—a compound belonging to the subpathway of purine metabolism—was tentatively identified. AICAR is an intermediate in the generation of inosine monophosphate that is capable of regulation of AMP-dependent protein kinase activity (AMPK). In isolated rodent muscle treated with AICAR, an increase in glucose transport by an insulin-independent mechanism has been observed [37], and this was associated with an increased translocation of the insulin-sensitive glucose transporter GLUT4 to sarcolemmal membranes in skeletal muscle [38]. In another study with isolated heart muscles incubated with AICAR, activation of AMP-dependent protein kinase activity, stimulation of glucose uptake, and translocation of the cardiomyocyte glucose transporter GLUT4 to the cell surface have been observed. AICAR treatment has also increased the phosphorylation of endothelial nitric oxide synthase [39]. In in vitro study on skeletal muscle from nondiabetic men, AICAR exposure has increased glucose transport in a dose-dependent manner [40]. These functions of AICAR suggest that this compound could increase glucose transport to working muscles and its use as a substrate for anaerobic glycolysis.

Another metabolite that deserves special attention, which fold change, especially in the women, was extremely huge, is 4-Hydroxynonenal. This compound is generated during reactions of the reactive nitrogen species (RNS) and reactive oxygen species (ROS) with polyunsaturated fatty acids (PUFAs). In this process, hydrogen is abstracted from the alpha carbon with the insertion of oxygen, which results in the generation of lipid peroxylradicals, and after that, these products are oxidized to lipid hydroperoxides. Consequently, many diverse products are generated, with 4-Hydroxynonenal in particular. It is a major product of decomposition of linoleic and arachidonic fatty acids. Therefore, membrane phospholipids, which have a high content of PUFAs (arachidonic, linoleic, linolenic, docosahexaenoic, and eicosapentaenoic acids) are extremely sensitive to attacks by ROS and RNS [41]. 4-Hydroxynonenal is highly diffusible and, therefore, it can spread beyond initial generation sites and can act as a stress signaling molecule [42]. The uncontrolled excessive production of 4-Hydroxynonenal can interfere with normal cellular signaling, react with lipids that contain an amino group, nucleic acids, and proteins, and consequently, it can lead to the development of many pathological conditions [43,44]. In particular, 4-Hydroxynonenal-protein adducts have been extensively investigated in diseases characterized by the pathogenic contribution of oxidative stress, such as cancer, neurodegenerative, chronic inflammatory, and autoimmune diseases [44]. However, on the other hand, recent observations on the role of 4-Hydroxynonenal in signaling have also shed new light on adaptive cytoprotective responses. It has been shown that activation of 4-Hydroxynonenal sensors stimulates the elimination of the damaged cellular components, prepares the cells for antioxidant defence, or even serves as protection of the organism by inducing apoptosis in severely injured cells [45,46]. Considering the present state of knowledge on the effects of 4-Hydroxynonenal, it is difficult to determine what consequences such a high generation of this compound in conditions of intense exercise combined with beetroot juice supplementation may have. 

## 4. Limitations of the Study

In our research, we devoted a lot of attention to the criteria for including the fencers into the study and monitoring their diet and physical activity. However, our study also has some limitations. Metabolomic analysis of the beetroot juice, which was given to the fencers, would be an excellent supplementation of the study. We also did not perform an analysis of the nitrite and nitrate concentrations excreted in the urine. Interpretation of the results obtained, especially those with regard to the neurotransmitters, may also result in some limitations due to the fact that these were urine samples that constituted the research material. An analysis of the brain levels of neurotransmitters can be performed in cerebrospinal fluid, but this is a highly invasive procedure. For this reason, alternative methods for estimating levels of neurotransmitters in the brain are being sought. For example, Audhya et al., in their studies using rat and human samples, have observed a significant correlation of a serotonin level in cerebrospinal fluid not only with the new method for measuring platelet serotonin level but also with levels in plasma and in the urine [47]. Therefore, urine samples may appear to be an alternative biological material for the study of neurotransmitters.

## 5. Conclusions

Our results shed new light on physiological mechanisms that are activated after the long-term supplementation of beetroot juice. We used the UPLC Q-TOF/MS system for the analysis of the urine samples taken 1 hour after the cardiovascular fitness test. It is also important that sampling this biological material is non-invasive and can be widely used in studies involving various exercise and supplementation protocols for a more complete understanding of the metabolomics effects of dietary nitrate. Overall, we observed significant changes in tyrosine and tryptophan metabolism mainly connected, bearing in mind the magnitude of the response, with such neurotransmitters as: Serotonin, noradrenaline, and adrenaline. This indicates that dietary nitrates may induce the activity of these neurotransmitters. High signal intensity from the other metabolites from the conversion of tyrosine, tryptophan, and arginine indicate that these amino acids were also partly used as an energy substrate and entered the tricarboxylic acid cycle. Furthermore, changes in signal intensity of bile acids and AICAR were observed, which could support the supply and use of substrates during an increased energy demand. Finally, an additional metabolite that deserves special attention, the fold change of which, especially in the women, was extremely huge, is 4-Hydroxynonenal—generated during reactions of the reactive nitrogen and oxygen species with polyunsaturated fatty acids. To our knowledge, it has been reported for the first time that the long-term beetroot juice supplementation induces such a kind of change in metabolism.

## 6. Materials and Methods 

### 6.1. Study Participants and Study Design 

For the current study, 10 female (age 22.6 ± 4.7 years, height 170.9 ± 8.4 cm) and 10 male (age 27.2 ± 5.4 years, height 185.9 ± 5.3 cm) fencers who train in fencing at least 10 hours a week and have been training for at least 3 years, were recruited. All the athletes competed at the international level. Exclusion criteria for participation in the study were as follows: The use of alternative diets, smoking, the occurrence of chronic diseases, taking dietary supplements or other ergogenic agents, as well as antibiotics and steroid or non-steroidal anti-inflammatory drugs, in the case of women also irregular menstruation. The study was conducted during the preparatory phase (from October to December). After receiving a detailed explanation of the study protocol, each participant provided written informed consent. The study was approved by the Ethics Committee of the Nofer Institute of Occupational Medicine (NR 05/2015). 

The study protocol in the women and men was the same, and included 2 stages. In the first stage lasting 4 weeks, the dietary recommendations were implemented (stage D) and after that in the second stage also lasting 4 weeks, the same dietary recommendations were implemented and additionally, the fencers received freeze-dried beetroot juice in the amount of 26 g per day (stage D&J). During each stage of the study, nutrition and physical activity were analyzed on 3 following days: A day with a general development training, a day with a fencing training, and a training-free day. In the women and men at baseline (B) and after each stage of the study, urine samples were collected. In addition, body composition and cardiovascular fitness (maximal oxygen consumption test - VO_2max_) were measured.

**Figure 4:**
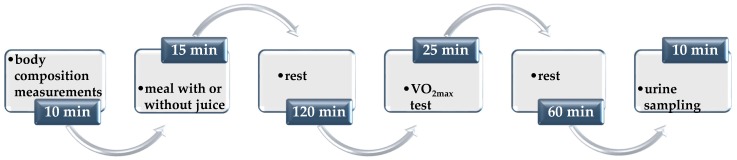
The protocol of laboratory measurements conducted in the group of female and male fencers after each stage of the study. The fencers after the first stage before the maximal oxygen consumption test (VO_2max_) consumed a meal with maltodextrin (26 g), whereas after the second stage, they consumed a meal with freeze-dried beetroot juice (26 g)—both dissolved in water.

At B and after the last day of each stage, the fencers, after having been fasting all night, visited a laboratory. After emptying their bladder, the body composition was measured and then the athletes consumed a meal with a specified composition: Toast bread (120 g), cold meat—sirloin (60 g), a banana (100 g), butter mix (24 g) and dissolved in 150 mL of water: Maltodextrin (26 g) or freeze-dried beetroot juice (26 g). The basic nutritional value of this meal was as follows: Energy—787 kcal, protein—23 g, total fat— 27 g, total carbohydrate—117 g. The fencers at B and after stage D before the VO_2max_ test consumed a meal with maltodextrin, whereas after D&J they consumed a meal with freeze-dried beetroot juice. The meal was consumed within 15 min, and 2 hours later, the VO_2max_ test was performed. The urine samples for the metabolomic analysis were collected 1 hour after the end of the VO_2max_ test. The protocol of the subsequent steps during the visit of the fencers in the laboratory is presented in Figure 4.

At baseline, the athletes received the dietary recommendations with individually calculated energy and nutrients values, together with a plan of meals. Total energy demand for each person was estimated on the basis of the filled-in physical activity logbook and energy cost of physical activities (Metabolic Equivalent of Task—MET), [48]. Nutritional recommendations for the athletes regarding protein intake [49] and for the general population regarding other nutrients [50] were incorporated into developing the personalized diet plans. A computer program Dieta 5 (Warsaw, Poland), was used to determine daily energy and nutrients intake. In the analysis of nutrients intake during the second stage of the study with freeze-dried beetroot juice supplementation, nutritional values of this portion of the juice were not incorporated into the daily nutrients intake. Two times at each stage the fencers filled in the 3-day dietary records of consumed food and beverages and the 3-day physical activity logbook. The information came from the following three days: a day with fencing training, a day with general training and a day free from training. Due to the fact that there were no significant differences in the level of physical activity and nutrient intake during the first stage of the study, the data collected twice was averaged. The collected data (dietary intake and physical activity) from the second stage of the study was also averaged. 

Weight of the fencers dressed in light bathing suits was measured to the nearest 10 g using a digital scale. Height was measured to the nearest 5 mm using a wall-mounted stadiometer. Body composition (fat-free mass—FFM; fat mass—FM) was measured using a bioimpedance analyzer BC418MA (Tanita, Tokyo, Japan) with a constant current frequency of 50 kHz and a system of 8 still, stainless-steel electrodes mounted on a platform. The measurements were performed according to the standard procedures [51].

VO_2max_ was measured using an incremental protocol [52] on the bicycle ergometer Ergoselect 200 (Ergoline GmbH, Bitz, Germany) and the ergospirometer Mes 2000 (MES, Cracow, Poland). Briefly, each participant was pedalling at a load of 50 W for 5 min with a self-selected speed as part of a warm-up. After that, the load increased by 50 watts every 3 min until volitional exhaustion, i.e., when a pedaling frequency of 60 revolutions per minute (rpm) could no longer be maintained. Respiratory gas exchange was measured breath-by-breath and heart rate was recorded. The average value of gas for each 30 s period was used for the analysis, and the highest VO_2_ in a 30 s period was defined as VO_2max_.

### 6.2. Preparation of the Freeze-Dried Beetroot Juice

Freeze-dried beetroot juice was prepared by a specialized company that has the authorization to produce this type of product. First, the raw beetroot (*Beta vulgaris var. rubra*) was peeled, grounded, and then centrifuged to separate the juice. After that, the juice was heated for 5 min at 100 °C and quickly chilled and frozen at −22 °C for 12 h. Then, the frozen juice was freeze-dried and this process lasted 20 h. At the end of this process, the product was ground into a powder and hermetically packed in portions of 26 g. Such a portion of freeze-dried beetroot juice was equivalent to 1 glass (200 mL) of fresh beetroot juice (before the process of freeze-drying).

We looked for information about the content of protein and amino acids in beetroot and in juice prepared from this vegetable in the available literature. The amount of protein in beetroot was very small—about 1.35 g/100 g [53] and this product had only a trace amount of L-Tryptophan (26.363 mg/100 g) and L-Tyrosine (40.720 mg/100 g), [54], whereas, the amount of protein in beetroot juice was 0 g/100 mL [55].

### 6.3. Metabolomics Analysis 

#### 6.3.1. Chemicals Applied

Formic acid LC-MS grade Sigma-Aldrich (St. Louis, MO, USA), acetonitrile, and isopropanol UHPLC-MS grade Chem-SolveTM S.Witko (Lodz, Poland) were applied. Ultra-high purity water was prepared by the R5 UV Hydrolab system (Wislina, Poland). Sodium formate calibration solution and leucine encephalin lock mass solution (Waters, Blackwood, UK) were prepared according to the manufacturer’s specifications.

#### 6.3.2. Methods of Metabolomics Analysis 

The urine samples collected after the VO_2max_ test were stored at −80 °C and prior to the UPLC-MS analysis, they were thawed, vortex mixed, and diluted with pre-chilled (4 °C) ultra-high purity water 1:1. After being vortex mixed (10 min), they were centrifuged at rpm for 15 min at 4 °C. The supernatants were subjected to the UPLC Q-TOF/MS system for analysis. A pooled “quality control” (QC) sample was prepared by mixing equal aliquots (50 μL) from the extracted samples for optimization of the chromatographic and TOF/MS conditions. QC samples were prepared separately from the group of all the women and after that from the group of all the men. 

The urine samples analysis was performed using the Waters Acquity™ Ultra Performance LC system (Waters Corp., Milford, USA) connected to a Synapt G2Si Q-TOF mass spectrometer (Waters MS Technologies, Manchester, UK) equipped with an electrospray (ESI) source (Waters, Manchester, UK). An ACQUITY UPLC HSS T3 column (1.8 um, 2.1 × 100 mm) was applied with a ACQUITY UPLC HSS T3 1.8 um, VanGuard Pre-Column 3/Pk 2.1 × 5 mm (Waters Corporation, Milford, NA, USA). The injection volume was 5 μL, and separation was performed at 0.4 mL/min and 50 °C. The mobile gradient phase was a mixture of 0.1% formic acid in water (A) and in acetonitrile (B). The gradient elution was performed in a following manner: 0–2 min 1% phase B; 2–8 min from 1% to 30% B; 8–11 min from 30% to 95% B; 11–13.5 min from 95% to 100% B; 13.5–16.0 min 100% B; 16.0–16.5 min from 100% to 1% B; 16.5–20.0 min 1% B.

All the tests were performed in ESI+ and ESI- ionization modes. Nitrogen gas was used as a cone and desolvation gas. The desolvation gas flow was set at 15 L/min at a temperature of 350 °C, the cone gas was set at 0.83 L/min, and the source temperature was set at 120 °C. In ESI+ ion mode, the capillary voltage was 3.2 kV, and in ESI- ion mode, the capillary voltage was 2.4 kV. All the data were acquired using the lock mass to ensure accuracy and reproducibility. Leucine enkephalin was used as the lock mass at a scan time 0.1 s, interval 30 s, and mass window ± 0.5 Da. The MS method was used for the data collection with a scan time of 0.2 s, data format centroid, and high-resolution mode. Prior to the analysis, the QC sample ran 7 times to test the stability of the instrument. During the analytical run, the QC sample was injected before and every 5th experimental sample to monitor the system consistency. The method for preparing the urine samples and procedure for their analysis were consistent with the protocol of urine metabolomics [56].

The collected MassLynx raw data files were converted using the DataBridge program into NetCDF format. In this format, the files were loaded into the XCMS Online program [57]. The default XCMS parameter set for UPLC—High Res (Waters) was used—the detailed methodology was published in the paper of Kozlowska et al. [58]. 

Potentially identified compounds obtained from the data processing in the XCMS Online were subjected to fragmentation using the average collision energy of 20 V in fast DDA mode. A QC sample was used to fragment the compounds, and the chromatographic and spectrometer parameters, except for collision energy, were the same as for the MS mode. Fast DDA parameters for MS mode were as follows: Range 90 to 1200 Da; scan time 0.1 s and for MS/MS mode: Range 50 to 1000 Da; select ion number 2; MS/MS switch off after 0.1 s; scan time 0.05 s. The resulting fragmentation spectra of the compounds were compared to the spectra in the HMDB (The Human Metabolome Database).

### 6.4. Statistical Analysis Methods

Normality distributions of the continuous data were inspected with the Shapiro-Wilk W test and presented as the mean ± SD. Differences in the continuous variables before and after the freeze-dried beetroot juice supplementation were assessed using the ANOVA with repeated measures and the post-hoc NIR Fisher test. Values of *p* < 0.05 were accepted as statistically significant. These statistical procedures were completed by means of Statistica (StatSoft Inc., Tulsa, OK, USA) version 13.1 software. The following statistical options were used in the XCMS program: The paired non-parametric Wilcoxon signed rank test, *p*-value threshold of 0.05, the fold change threshold of 1.2, and median fold change normalization. A *q*-value threshold of < 0.1 was used to remove any *p*-values (up to a 95% confidence) that could have been false positives [59]. Due to the fact that the studied metabolomic parameters depended to a large extent on gender, the statistical analysis of the obtained results was performed in the group of women and men separately (before vs. after the beetroot juice supplementation). 

## Figures and Tables

**Table 1:** Anthropometric parameters, physical activity level, and cardiovascular fitness at baseline and after consecutive stages of the study in the women (*n* = 10) and men (*n* = 10).

Studied Group/Variable	Stages of the Study			*p* Value	
	B	after D	after D&J	*p* ^a^	*p* ^b^
**Women**					
Weight (kg)	63.6 ± 8.03	63.5 ± 8.3	63.6 ± 8.6	0.7434	0.8011
FM (kg)	16.9 ± 4.0	16.5 ± 5.1	16.1 ± 4.8	0.3390	0.2140
FFM (kg)	46.7 ± 5.8	47.0 ± 4.9	47.5 ± 5.3	0.5625	0.1455
PA - FT	1.87 ± 0.24	1.88 ± 0.14	1.88 ± 0.18	0.8737	0.9540
PA - GT	1.59 ± 0.20	1.71 ± 0.19	1.76 ± 0.22	0.1701	0.5044
PA - FD	1.36 ± 0.11	1.41 ± 0.14	1.49 ± 0.16	0.3550	0.2125
VO_2max_(mL/kg/min)	39.3 ± 4.8	39.4 ± 4.8	42.8 ± 4.7	0.6681	**0.0106**
**Men**					
Weight (kg)	82.5 ± 11.6	82.5 ± 11.9	82.7 ± 11.4	0.8206	0.5631
FM (kg)	15.0 ± 4.8	14.3 ± 4.5	14.0 ± 4.3	0.0676	0.5053
FFM (kg)	67.6 ± 8.2	68.2 ± 8.5	68.7 ± 8.4	0.1197	0.2104
PA - FT	2.10 ± 0.33	1.96 ± 0.28	1.98 ± 0.29	**0.0275**	0.7611
PA - GT	1.80 ± 0.30	1.83 ± 0.23	1.88 ± 0.31	0.7113	0.6274
PA - FD	1.53 ± 0.18	1.43 ± 0.16	1.42 ± 0.16	0.1154	0.8876
VO_2max_(mL/kg/min)	46.0 ± 7.8	45.3 ± 9.3	48.5 ± 10.3	0.7276	**0.0234**

Values are presented as mean ± SD; *p* ^a^ = B versus D; *p* ^b^ = D versus D&J; bold values denote statistical significance at the *p* < 0.05 level; B—baseline parameters; D—the first stage of study (implementation of dietary recommendations); D&J—the second stage of study (implementation of dietary recommendations and beetroot juice supplementation); FM— fat mas; FFM— fat free mass; PA-FT—mean physical activity level in days with fencing training; PA-GT—mean physical activity level in days with general training; PA-FD— mean physical activity level in free day; VO_2max_—maximum rate of oxygen uptake (parameter of cardiovascular fitness).

**Table 2:** The daily energy and selected nutrients intake in the studied groups of fencers during consecutive stages of the study in the women (*n* = 10) and men (*n* = 10).

Variable		Women			Men	
	during D	during D&J	*p* ^a^	during D	during D&J	*p* ^b^
Energy (kcal)	2152 ± 248	2136 ± 219	0.6862	2498 ± 378	2591 ± 401	**0.0262**
Protein (g)	96.6 ± 10.8	93.2 ± 8.3	0.5906	109.9 ± 42.8	98.0 ± 24.3	0.0713
Fat (g)	87.3 ± 11.2	82.6 ± 14.0	0.1508	94.9 ± 10.0	102.2 ± 12.9	**0.0278**
Total carbohydrates (g)	266.5 ± 37.9	275.5 ± 25.4	0.1436	302.4 ± 56.2	307.2 ± 69.1	0.4240
Potassium (mg)	3854.7 ± 596.5	3881.8 ± 575.4	0.8776	4174.1 ± 1398.1	4013.7 ± 979.9	0.3680
Calcium (mg)	975.3 ± 366.7	984.6 ± 333.3	0.8466	1003.4 ± 315.0	939.1 ± 235.4	0.1916
Magnesium (mg)	391.8 ± 61.5	384.7 ± 40.8	0.7098	435.7 ± 141.2	442.5 ± 111.5	0.5629
Phosphorus (mg)	1742.7 ± 231.1	1703.1 ± 213.6	0.5126	2064.2 ± 742.8	1981.4 ± 603.8	0.1791
Iron (mg)	13.29 ± 1.50	12.99 ± 1.54	0.5637	14.98 ± 3.81	14.89 ± 2.72	0.8527
Zinc (mg)	12.61 ± 1.88	12.54 ± 2.01	0.8669	14.73 ± 3.95	14.30 ± 3.19	0.2759
Vitamin A equ. (µg)	1226.4 ± 279.5	1195.9 ± 287.1	0.7742	1523.5 ± 495.5	1400.4 ± 505.7	0.2479
Vitamin E equ. (mg)	12.60 ± 2.36	12.64 ± 3.07	0.9669	13.98 ± 4.30	15.22 ± 3.44	0.1317
Vitamin C (mg)	124.0 ± 57.1	124.4 ± 38.4	0.2125	138.1 ± 73.1	148.3 ± 57.4	0.4616
Folate equ. (µg)	347.1 ± 62.4	350.1 ± 62.7	0.8371	380.9 ± 116.4	377.1 ± 83.9	0.7929
Vitamin B_1_ (mg)	1.38 ± 0.21	1.45 ± 0.21	0.5262	1.68 ± 0.62	1.55 ± 0.36	0.2405
Vitamin B_2_ (mg)	2.07 ± 0.23	2.04 ± 0.20	0.6358	2.23 ± 0.92	2.15 ± 0.73	0.2332
Vitamin B_6_ (mg)	2.23 ± 0.30	2.19 ± 0.33	0.6962	2.91 ± 1.21	2.78 ±0.98	0.1912
Vitamin B_12_ (µg)	4.52 ± 1.28	3.96 ± 0.80	0.2190	7.83 ± 4.17	6.64 ± 4.06	**0.0159**
Niacin equ. (mg)	22.11 ± 5.17	20.65 ± 5.66	0.1914	29.19 ± 9.91	28.43 ± 9.23	0.4897

values are presented as mean ± SD; *p* ^a^— D&J versus D in group of women; *p* ^b^— D&J versus D in group of men; bold values denote statistical significance at the *p* < 0.05 level; D—the first stage of study (implementation of dietary recommendations); D&J—the second stage of study (implementation of the dietary recommendations and beetroot juice supplementation); equ.—equivalent.

## Supplementary Materials

The following are available online at https://www.mdpi.com/2218-1989/10/3/100/s1, Table S1. General characteristics of potential biomarkers tentatively identified in the negative and positive ESI modes in the group of the women: Stage D&J (implementation of dietary recommendations and beetroot juice supplementation) versus stage D (implementation of dietary recommendations without beetroot juice supplementation), Table S2. General characteristics of potential biomarkers tentatively identified in the negative and positive ESI modes in the group of men: stage D&J (implementation of dietary recommendations and beetroot juice supplementation) versus stage D (implementation of dietary recommendations without beetroot juice supplementation).
