# Supplementary material for: An Untargeted Metabolomics Approach to Investigate the Metabolic Effect of Beetroot Juice Supplementation in Fencers—A Preliminary Study
Source: Metabolites. 2020 Mar 11;10(3):100. doi: 10.3390/metabo10030100 (PMC7143097; doi:10.3390/metabo10030100)
Supplement: Supplementary file 1 [file metabolites-10-00100-s001.pdf]

Table S1. General characteristics of potential biomarkers tentatively identified in the negative and positive ESI modes in the group of the women: stage D&J (implementation of dietary recommendations and beetroot juice supplementation) versus stage D (implementation of dietary recommendations without beetroot juice supplementation)

Metabolites (ID in HMDB)	ESI mode	RT	Molecular weight	m/z	Adducts	m/z diff.	Fit (%)	Dysregu-lation	FC	p-value	Subpathway(s) involved
Norepinephrine (HMDB0000216)	-	0.83	169.0739	168.0660	M-H[-]	-0.0006	0.97	UP	1.3	0.006	noradrenaline and adrenaline deg.
Vanilpyruvic acid (HMDB0011714)	-	6.61	210.0528	209.0453	M-H[-]	-0.0003	0.97	UP	2.3	0.006	noradrenaline and adrenaline deg.
3-Methoxy-4-hydroxyphenyl-glycolaldehyde (HMDB0004061)	+	4.95	182.0579	200.0922	M+NH3+H[1+]	0.0005	0.86	UP	1.6	0.002	noradrenaline and adrenaline deg.
3,4-Dihydroxymandelic acid (HMDB0001866)	-	1.93	184.0372	229.0344	M+HCOO[-]	-0.0004	0.78	UP	12.3	0.002	noradrenaline and adrenaline deg.
Vanillylmandelic acid (HMDB0000291)	-	4.34	198.0528	197.0452	M-H[-]	-0.0004	0.80	UP	6.3	0.004	noradrenaline and adrenaline deg.
Vanylglycol (HMDB0001490)	-	5.71	184.0736	165.0551	M-H2O-H[-]	-0.0006	0.88	UP	2.0	0.004	noradrenaline and adrenaline deg.
Leucodopachrome (HMDB0004067)	-	5.76	195.0532	176.0345	M-H2O-H[-]	-0.0008	0.93	UP	6.8	0.004	L-dopa and L-dopachrome bios.
5,6-Dihydroxyindole (HMDB0004058)	+	4.66	149.0477	150.0555	M+H[1+]	0.0005	0.80	UP	14.1	0.006	L-dopa and L-dopachrome bios.
5-Hydroxyindoleacetic acid (HMDB0000763)	-	5.51	191.0582	236.0558	M+HCOO[-]	-0.0001	0.92	UP	55.3	0.002	serotonin and melatonin deg.
N-Acetylserotonin (HMDB0001238)	+	4.21	218.1055	110.0605	M+2H[2+]	0.0005	0.91	UP	100.2	0.004	serotonin and melatonin deg.
Acetyl-N-formyl-5-methoxy-kynurenamine (HMDB0004259)	-	4.06	264.1110	309.1090	M+HCOO[-]	0.0003	0.98	UP	1.5	0.002	serotonin and melatonin deg.
N-Acetyl-5-methoxykynuramine (HMDB0062497)	-	4.87	236.1161	281.1141	M+HCOO[-]	0.0004	0.98	UP	2.3	0.010	serotonin and melatonin deg.
5-Hydroxytryptophol (HMDB0001855)	+	3.43	177.0790	160.0763	M-H2O+H[1+]	0.0006	0.88	UP	6.8	0.002	serotonin and melatonin deg.
Indoleacetaldehyde (HMDB0001190)	+	3.43	159.0684	160.0763	M+H[1+]	0.0006	0.93	UP	6.8	0.002	L-tryptophan deg.
Oxoadipic acid (HMDB0000225)	-	2.00	160.0372	205.0349	M+HCOO[-]	0.0001	0.83	UP	1.5	0.006	L-tryptophan deg.
4-Acetamidobutanoic acid (HMDB0003681)	-	4.07	145.0739	108.0449	M-2H2O-H[-]	-0.0005	0.89	UP	6.9	0.002	putrescine deg. III

N4-Acetylaminobutanal (HMDB0004226)	-	3.72	129.0790	188.0925	M+CH3COO[-]	0.0002	0.74	UP	1.5	0.010	putrescine deg. III
D-Xylose (HMDB0000098)	-	4.30	150.0528	113.0240	M-2H2O-H[-]	-0.0003	0.87	UP	1.8	0.006	D-xylose deg. I
Pyridoxine (HMDB0000239)	+	1.24	169.0739	152.0711	M-H2O+H[1+]	0.0005	0.79	UP	19.9	0.002	pyridoxal 5'-phosphate salvage
Pyridoxal (HMDB0001545)	-	4.51	167.0582	148.0399	M-H2O-H[-]	-0.0005	0.83	UP	54.5	0.006	pyridoxal 5'-phosphate salvage
Ascorbic acid (HMDB0000044)	-	4.30	176.0321	175.0245	M-H[-]	-0.0003	0.94	UP	2.2	0.006	ascorbate recycling
4-Hydroxynonenal (HMDB0004362)	+	6.09	156.1150	195.0772	M+K[1+]	-0.0006	0.96	UP	714.9	0.002	4-hydroxy-2-nonenal detoxification
AICAR (HMDB0001517)	+	4.08	338.0628	356.0981	M+NH3+H[1+]	0.0016	0.91	UP	2.6	0.004	purine nucleotide bios.

ESI - electrospray ionization; Fit(%) - score that indicates the percentage of peaks in the library MS/MS spectrum found in the query spectrum; HMDB – Human Metabolome Database; RT – retention time; m/z - ratio of the weight of the compound to the electric charge; m/z diff. – m/z difference; FC – fold change: UP - direction of change D&J vs. D; deg. – degradation; bios. - biosynthesis

Table S2. General characteristics of potential biomarkers tentatively identified in the negative and positive ESI modes in the group of men: stage D&J (implementation of dietary recommendations and beetroot juice supplementation) versus stage D (implementation of dietary recommendations without beetroot juice supplementation)

**Metabolites (ID in HMDB)**	**ESI mode**	**RT**	**Molecular weight**	**m/z**	**Adducts**	**m/z diff.**	**Fit (%)**	**Dysregu-lation**	**FC**	**p-value**	**Subpathway(s) involved**
Norepinephrine (HMDB0000216)	-	0.84	169.0739	168.0662	M-H[-]	-0.0004	0.97	UP	2.2	0.004	noradrenaline and adrenaline deg.
3,4-Dihydroxymandelic acid (HMDB0001866)	-	1.91	184.0372	229.0350	M+HCOO[-]	0.0002	0.78	UP	18.5	0.002	noradrenaline and adrenaline deg.
3,4-Dihydroxyphenylglycol (HMDB0000318)	-	5.51	170.0579	133.0292	M-2H2O-H[-]	-0.0003	0.78	UP	1.8	0.004	noradrenaline and adrenaline deg.
3,4-Dihydroxymandelaldehyde (HMDB0006242)	+	9.60	168.0423	186.0765	M+NH3+H[1+]	0.0005	0.86	UP	1.5	0.002	noradrenaline and adrenaline deg.
Vanillactic acid (HMDB0000913)	-	5.51	212.0685	193.0503	M-H2O-H[-]	-0.0002	0.90	UP	1.8	0.004	noradrenaline and adrenaline deg.
Vanillylmandelic acid (HMDB0000291)	-	4.35	198.0528	197.0450	M-H[-]	-0.0006	0.80	UP	6.5	0.002	noradrenaline and adrenaline deg.
5,6-Dihydroxyindole (HMDB0004058)	+	4.64	149.0477	150.0556	M+H[1+]	0.0007	0.80	UP	8.6	0.002	L-dopa and L-dopachrome bios.
Leucodopachrome (HMDB0004067)	-	5.75	195.0532	176.0346	M-H2O-H[-]	-0.0006	0.93	UP	4.5	0.002	L-dopa and L-dopachrome bios.
4a-Hydroxytetrahydrobiopterin (HMDB0002281)	+	3.48	257.1124	280.1015	M+Na[1+]	-0.0002	0.96	UP	3.2	0.002	catecholamine bios.
4-Fumarylacetoacetic acid (HMDB0001268)	-	1.21	200.0321	245.0309	M+HCOO[-]	0.0012	0.94	UP	7.1	0.002	L-tyrosine deg. I
5-Hydroxyindoleacetic acid (HMDB0000763)	-	5.51	191.0582	236.0562	M+HCOO[-]	0.0003	0.92	UP	66.9	0.002	serotonin and melatonin deg.
N-Acetylserotonin (HMDB0001238)	+	4.19	218.1055	110.0606	M+2H[2+]	0.0005	0.91	UP	44.3	0.002	serotonin and melatonin deg.
N-Acetylserotonin glucuronide (HMDB0060833)	-	5.31	394.1376	196.0610	M-2H[2-]	-0.0006	0.73	UP	2.6	0.004	serotonin and melatonin deg.
N-Acetyl-5-methoxykynuramine (HMDB0062497)	+	3.53	236.1161	275.0790	M+K[1+]	0.0002	0.85	UP	2.1	0.002	serotonin and melatonin deg.
Tryptophanol (HMDB0003447)	+	4.04	161.0841	145.0654	M-NH3+H[1+]	0.0006	0.83	UP	3.3	0.002	L-tryptophan deg.
4-Acetamidobutanoic acid (HMDB0003681)	-	4.05	145.0739	108.0451	M-2H2O-H[-]	-0.0004	0.89	UP	11.6	0.002	putrescine deg. III

N4-Acetylaminobutanal (HMDB0004226)	-	4.23	129.0790	174.0768	M+HCOO[-]	0.0002	0.74	UP	1.3	0.004	putrescine deg. III
D-Xylose (HMDB0000098)	-	4.31	150.0528	113.0240	M-2H2O-H[-]	-0.0004	0.87	UP	2.2	0.002	D-xylose deg. I
Pyridoxine (HMDB0000239)	+	1.22	169.0739	152.0713	M-H2O+H[1+]	0.0007	0.79	UP	10.5	0.002	pyridoxal 5'-phosphate salvage
Pyridoxal (HMDB0001545)	-	4.54	167.0582	166.0502	M-H[-]	-0.0007	0.83	UP	3.8	0.002	pyridoxal 5'-phosphate salvage
Ascorbic acid (HMDB0000044)	-	4.65	176.0321	175.0246	M-H[-]	-0.0002	0.94	UP	1.9	0.002	ascorbate recycling
4-Hydroxynonenal (HMDB0004362)	+	6.08	156.1150	195.0773	M+K[1+]	-0.0005	0.96	UP	20.1	0.002	4-hydroxy-2-nonenal detoxification
Chenodeoxycholic acid glycine conjugate (HMDB0000637)	-	10.29	449.3141	448.3065	M-H[-]	-0.0004	1.00	UP	1.6	0.002	bile acid deg.
Glycocholic acid (HMDB0000138)	-	10.19	465.3090	446.2905	M-H2O-H[-]	-0.0007	1.00	UP	2.0	0.004	bile acid deg.

ESI - electrospray ionization; Fit(%) - score that indicates the percentage of peaks in the library MS/MS spectrum found in the query spectrum; HMDB – Human Metabolome Database; RT – retention time; m/z - ratio of the weight of the compound to the electric charge; m/z diff. – m/z difference; FC – fold change: UP - direction of change D&J vs. D; deg. – degradation; bios. - biosynthesis
